# Inhibition of Angiogenesis, Fibrosis and Thrombosis by Tetramethylpyrazine: Mechanisms Contributing to the SDF-1/CXCR4 Axis

**DOI:** 10.1371/journal.pone.0088176

**Published:** 2014-02-05

**Authors:** Xiaoxiao Cai, Zhao Chen, Xueke Pan, Lei Xia, Pei Chen, Ying Yang, Huan Hu, Jing Zhang, Kaijing Li, Jian Ge, Keming Yu, Jing Zhuang

**Affiliations:** State Key Laboratory of Ophthalmology, Zhongshan Ophthalmic Center, Sun Yat-sen University, GuangZhou, GuangDong, P. R. China; Max-Delbrück Center for Molecular Medicine (MDC), Germany

## Abstract

**Background:**

Tetramethylpyrazine (TMP) is one of the active ingredients extracted from the Chinese herb Chuanxiong, which has been used to treat cerebrovascular and cardiovascular diseases, pulmonary diseases and cancer. However, the molecular mechanisms underlying the actions of TMP have not been fully elucidated. In a previous study we showed that TMP-mediated glioma suppression and neural protection involves the inhibition of CXCR4 expression. The SDF-1/CXCR4 axis plays a fundamental role in many physiological and pathological processes. In this study, we further investigated whether the regulation of the SDF-1/CXCR4 pathway is also involved in the TMP-mediated inhibition of neovascularization or fibrosis and improvement of microcirculation.

**Methodology/Principal Findings:**

Using a scratch-wound assay, we demonstrated that TMP significantly suppressed the migration and tubule formation of the human umbilical vein endothelial cell line ECV304 in vitro. The expression of CXCR4 in ECV304 cells is notably down-regulated after TMP treatment. In addition, TMP significantly suppresses corneal neovascularization in a rat model of corneal alkali burn injury. The expression of CXCR4 on days 1, 3 and 7 post-injury was determined through RT-PCR analysis. Consistent with our hypotheses, the expression of CXCR4 in the rat cornea is significantly increased with alkali burn and dramatically down-regulated with TMP treatment. Moreover, TMP treatment significantly attenuates bleomycin-induced rat pulmonary fibrosis, while immunofluorescence shows a notably decreased amount of CXCR4-positive cells in the TMP-treated group. Furthermore, TMP significantly down-regulates the expression of CXCR4 in platelets, lymphocytes and red blood cells. Whole-blood viscosity and platelet aggregation in rats are significantly decreased by TMP treatment.

**Conclusions:**

These results show that TMP exerts potent effects in inhibiting neovascularization, fibrosis and thrombosis under pathological conditions; thus, the underlying mechanism of TMP might partially contribute to the down-regulation of CXCR4.

## Introduction

Chuanxiong (Ligusticum wallichi Franchat) was first described in the Chinese traditional medicine book Shennong Bencaojing (a guide to Traditional Chinese Medicine), written in 200 BC. Chuanxiong is used in many clinical treatments, including those for ischemia, cerebral infarction and degenerative diseases of the central nervous system (Alzheimer's disease, Parkinson's disease and multiple sclerosis); myocardial and pulmonary fibrosis; and tumors, with mild side effects [1]–[6]. This herbal supplement can significantly attenuate platelet aggregation and thrombus formation, which improves whole-blood viscosity [7]–[8]. The bioactive component of chuanxiong, 2,3,5,6-tetramethylpyrazine (TMP), was first extracted in 1973 [9]. The soluble salts tetramethylpyrazine hydrochloride (TMPH) and TMP phosphate have been widely used in clinical treatments, including injections and oral tablets. According to the China Food and Drug Administration, there are currently 196 TMP-containing products clinically used in China [10].

Despite the wide application of TMP, there is no consensus among scholars regarding the mechanisms underlying this compound. Although accumulating evidence has identified TMP as a Ca^2+^ antagonist [11]–[12], its target gene remains unclear. Tan et al. reported that TMP significantly prevented lipid peroxidation and necrosis in neuronal cells, potentially by eliminating oxygen-free radicals [13]. The application of 50 µM TMP protected 80% of retinal neurocytes from H_2_O_2_-induced cell death in vitro. Moreover, using a glioma-neuronal co-culturing system, Wang et al. confirmed that TMP inhibited the viability of glioma cells while protecting hippocampal neurons and demonstrated that TMP promotes the regression of malignant gliomas in vivo [14]. In previous studies, we first demonstrated that TMP protects cerebral neurocytes and inhibits glioma cells by down-regulating the expression of the chemokine receptor CXCR4 [15]. The TMP-mediated down-regulation of CXCR4 in cerebral neurocytes inhibits somatic Ca2+ increase, decreases glutamate release from glial cells, and effectively inhibits the viability and migration of cultured C6 glioma cells, which induces neural protection and the suppression of C6 gliomas. Therefore, TMP might be a potential therapeutic candidate for the treatment of resistant malignant gliomas.

CXCR4, a 7-transmembrane spanning G protein-coupled receptor, is the only known receptor for SDF-1, which is characterized by its ability to induce cell invasion, locomotion, extravasation, directional migration, homing, and cell survival [16]–[18]. The SDF-1/CXCR4 axis is involved in the pathogenesis of several diseases, such as HIV, cancer, pathological angiogenesis and myocardial and pulmonary fibrosis [19]. For example, CXCR4 promotes angiogenesis in normal tissues, such as the cornea and retina, under pathological conditions, and in tumors. Unoki et al. showed that CXCR4 activated tip cells and microglia, resulting in retinal angiogenesis [20]. In a previous study, we also demonstrated that CXCR4 boosted the migration and tube formation of human retinal microvascular endothelial cells [21]. Moreover, Mehrad et al. reported the pharmacological inhibition of the CXCR4/CXCL12 biological axis in human fibrocytes and reduction of the magnitude of pulmonary fibrosis in vivo [22]. The CXCR4 antagonist, AMD3100, decreased CXCR4 expression and significantly attenuated pulmonary fibrosis in rats [23].

Furthermore, previous studies have demonstrated that CXCR4 regulates platelet aggregation. Dubois et al. reported that in platelets, CXCR4 interacts with bile salt-dependent lipase (BSDL) and modulates thrombus formation in mice and humans [24]. In a mouse thrombosis model, when CXCR4 was antagonized with AMD3100, the accumulation of BSDL was inhibited and thrombus size was reduced. In addition, the short-term administration of AMD3100 significantly improved blood restoration during the acute phase of ischemia in normal and diabetic mouse hindlimb ischemia models (25). Therefore, CXCR4 is a therapeutic target for many diseases in the circulatory system.

Based on the evidence described above, we hypothesized that the modulation of the SDF-1/CXCR4 axis might be involved in the TMP-mediated inhibition of pathological angiogenesis and the alleviation of pulmonary fibrosis and anti-thrombosis effects. Here, we established two animal models to test this hypothesis: a rat model of corneal alkali burn and a rat model of bleomycin-induced lung fibrosis. We demonstrated that TMP inhibits corneal neovascularization (NV) induced through alkali burn. CXCR4 expression is significantly increased in the rat cornea after alkali injury compared with that in the control group and subsequently dramatically down-regulated with TMP treatment. Moreover, TMP improves the whole-blood flow of SD rats after 3 days of treatment by decreasing blood viscosity and inhibiting platelet aggregation. TMP significantly down-regulates the expression of CXCR4 in platelets, lymphocytes and red blood cells. Therefore, these data suggest that the down-regulation of CXCR4 might be involved in the mechanisms of TMP treatment in clinical therapy.

## Results

### TMP blocks angiogenesis in vitro

**Figure 1:**
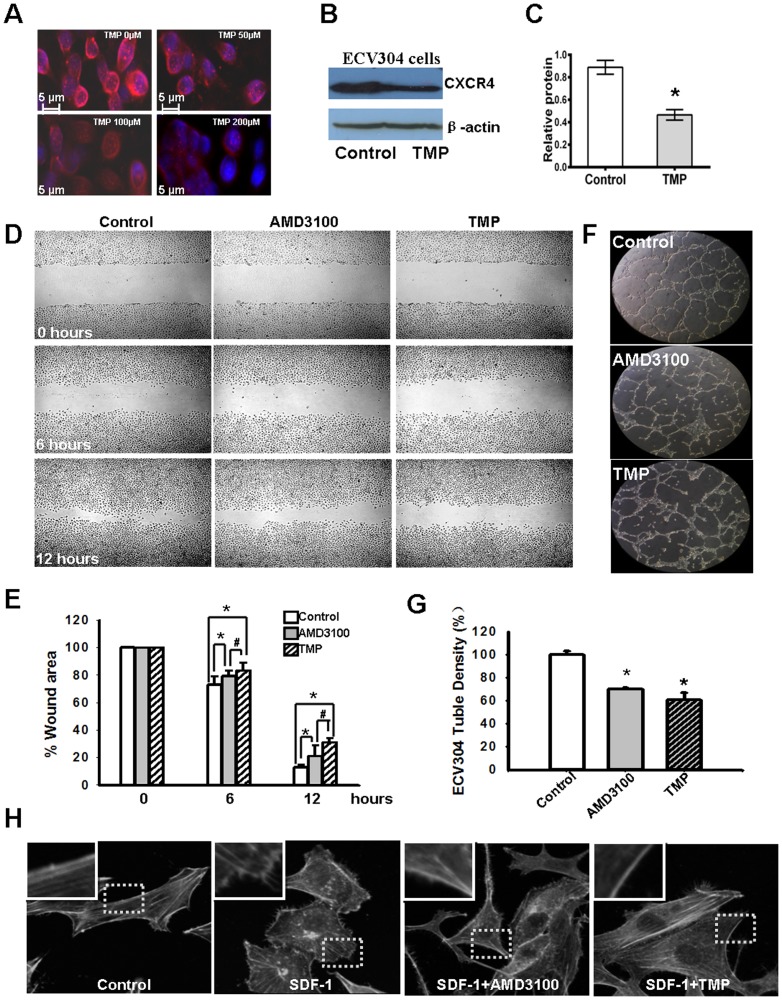
TMP blocks angiogenesis in vitro. (A) Immunocytofluorescence shows that the expression of CXCR4 in ECV304 cells (red) is down-regulated by TMP in a dose-dependent manner. (B) Western blot analysis indicates that TMP treatment down-regulated CXCR4 expression in ECV304 cells. (C) The relative expression of CXCR4 in ECV304 cells was quantified through densitometry. The expression of CXCR4 in ECV304 cells treated with TMP is significantly lower than that in control cells (0.825± 0.0617 and 0.423±0.0462, respectively, P<0.05). (D) The scratch wound-healing assay with ECV304 cells. The cells were seeded onto six-well plates in the presence of 100 µM of TMP, 10 µg/mL AMD3100 or PBS for 48 h and scratched using a sterile pipette tip to remove cells with two perpendicular linear scratches. Representative phase contrast images show wound-induced ECV304 cell migration for 6 h and 12 h. Original magnification, ×20. (E) Relative quantitation of ECV304 cell migration for 6 h and 12 h after scratch wounding. The data indicated that TMP significantly decreases the migration of ECV304 cells compared to the control, and meanwhile the inhibition of cell migration by TMP is more effective than that of AMD3100. (F) Microscopic photographs of tubule formation on Matrigel are shown. There was a significant reduction in ECV 304 cells tubule connections in the presence of TMP and AMD3100 as compared with control of PBS. (G) The tubule formation rates are presented in histograms. (H) TMP inhibits the cytoskeleton rearrangement of ECV304 cells stimulated by SDF-1(100 nM), and compared that with the effect of AMD3100 (10 µg/mL). The photomicrographs captured by a confocal microscope shows that the pseudopodia protrusion formation immediately after the stimulated by SDF-1, contains a large number of filopodia-like structures and it was inhibited by TMP and AMD3100. Original magnification, ×100. The error bars represent the s.d. of the mean. These results are representative of experiments performed at least three times. The asterisks represent statistically significant differences between TMP-treated cells, AMD3100-treated cells and the control, # represent statistically significant differences between AMD3100 and TMP (*p<0.005, #p<0.01).

Because the SDF-1/CXCR4 interaction plays a pivotal role in angiogenesis, we first examined CXCR4 expression in ECV304 cells after treatment with TMP at different concentrations in vitro(0, 50, 100, 200 µM). As shown in Fig. 1A, the expression of CXCR4 in ECV304 cells is down-regulated in a dose-dependent manner after TMP treatment. At 48 hours after treatment, the whole cell lysates were extracted for western blot assay. For quantification of the immunoblots, the relative intensities of the bands were quantified through densitometry, and the results were normalized to the β-actin levels. The relative quantification of CXCR4 expression in ECV304 cells treated with TMP was significantly lower than that in the control (0.825±0.0617 and 0.423±0.0462, respectively, P<0.05) (Fig. 1B, C). These results are consistent with those of previous reports, showing that TMP effectively down-regulates CXCR4 expression in C6 cells [15], [26].

The SDF-1/CXCR4 interaction is involved in cell migration and tubule formation [21]. The effect of TMP (100 µM) on cell migration was evaluated using a scratch-wound assay, and the results were compared to those from the cells treated with AMD3100 (10 µg/mL), a specific inhibitor of CXCR4. Wounds were artificially made in ECV304 cells after 48 h pre-treatment of TMP, AMD3100 or PBS. After a wound created, rapid proliferation of ECV304 cells in the border zones generated simultaneous close capture of the wounded edges. As shown in Fig. 1D, E, more remaining wound area was observed under microscopy at 6 h and 12 h after blocking CXCR4 by TMP and AMD3100. Moreover, blocking CXCR4 by TMP could significantly reduce the migration of ECV304 compared with that of AMD3100. Furthermore, tubule formation assays were used to measure the physiological effects of TMP on ECV304 cells. After treatment with 100 µM TMP, 10 µg/mL AMD3100 or PBS for 48 h, ECV304 cells form tubules within 24 h of culturing on Matrigel (Fig. 1F). TMP's effect on endothelial cell tubule formation is normalized by control which was treated by PBS, and compared to those from cells treated with AMD3100. With TMP or AMD3100 treatment ECV304 cells show markedly reduces tubule formation and do not develop vascular networks on Matrigel. Fig. 1G shows the quantification of tubule formation as measured using microscopy. TMP treatment eliminates capillary-like tubules and significantly attenuates tubule formation (P = 0.021).

Dynamic rearrangement of the cytoskeleton is a prerequisite for motility and migration of the cells [27]. To further demonstrate TMP effect on the axle of CXCR4/SDF-1, we thus performed actin-polymerization assay to examine if TMP alter the cytoskeleton structure of ECV304 cells stimulated by SDF-1, a known ligand for CXCR4. Cells were serum starved overnight, in the absence of TMP (100 µM), AMD3100 (10 µg/mL) or PBS, then stimulated with or without SDF-1(100 nM) for 15 minutes. Representative images of ECV 304 cells indicate that SDF-1 would cause micro filaments rearrangement along with filopodia-like structure formation in comparison to the control cells (Fig. 1H). Interestingly, the F-actin cytoskeleton remodeling induced by SDF-1 was counteracted by TMP or AMD3100 pre-treatment. These observations led to the speculation that TMP might inhibit angiogenesis through the regulation of SDF-1/CXCR4 signaling in vitro.

### TMP inhibits corneal neovascularization (NV) through alkali burn

**Figure 2:**
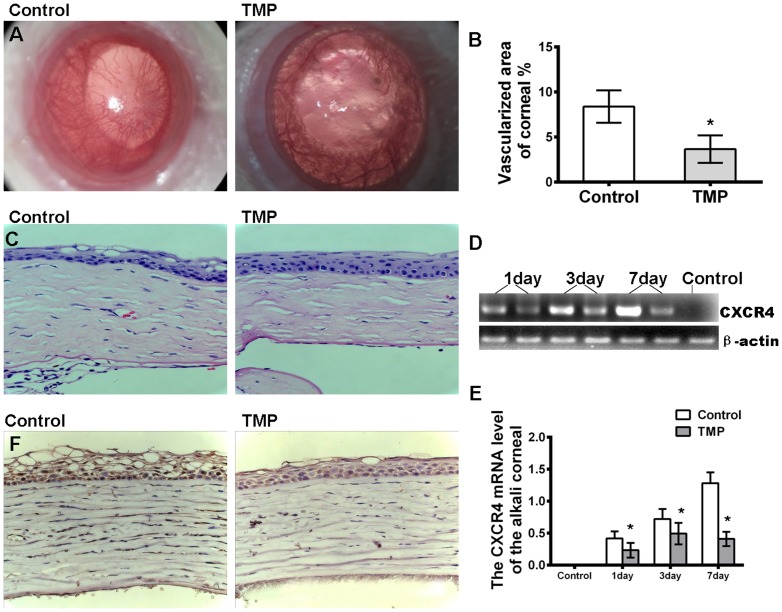
Effects of TMP on corneal NV. (A) Alkali-induced corneal neovascularization is suppressed through TMP treatment. Photos were taken at day 28. (B) The bar graph represents the mean and SE of the blood vessel within the microscopic fields from 15 eyes. (C) The hematoxylin and eosin staining of alkali-burned corneas at 28 days post-injury shows that stromal inflammation and edema (evaluated by the stromal thickness) are reduced in the corneas of the animals in the TMP-treated group compared with those in the control group. (D) The RT-PCR analysis of CXCR4 mRNA in the cornea post-injury. These results are representative of experiments performed at least three times. (E) The relative level of CXCR4 mRNA was quantified through densitometry. (F) Immunohistochemical staining indicates that CXCR4 (brown) is reduced in the cornea at 7 days after TMP treatment. The asterisks represent statistically significant differences between TMP and the control (*p<0.001).

To confirm the bioactivity of TMP on angiogenesis, a rat model of corneal alkali burn injury was established to investigate the effects of TMP on angiogenesis in vivo (Fig. 2A). The severity of corneal edema, percentage of neovascularization and length of the longest neovascular vessel in the alkali-burned cornea was quantified to evaluate the effect of TMP on corneal neovascularization as described in the Material and Methods. As shown in fig. 2B, the percentage of the neovascularized cornea increases over time in both groups. NV in rats treated with TMP is obviously suppressed compared with that in the PBS-treated group after alkali-burned injury. The relative quantification of corneal neovascularization is significantly lower in the TMP-treated group than in the control group (8.46±2.54 and 2.22±0.56, respectively) (Fig. 2B). The hematoxylin and eosin staining of alkali-burned corneas at day 28 post-injury showed that stromal inflammation and edema (assessed by the stromal thickness) are restrained to the periphery in the corneas of the animals in the TMP-treated group compared with those in the control group (Fig. 2C). The corneal epithelium in the control group is disorganized. The corneal stroma is thicker and more swollen than that in the TMP group (Fig. 2C). To determine whether CXCR4 is involved in the TMP-mediated reduction of NV, the expression of CXCR4 on days 1, 3 and 7 post-injury was determined through RT-PCR analysis. Consistent with our speculations, the level of CXCR4 mRNA is significantly increased after alkali burn compared with the normal cornea and dramatically down-regulated in rats treated with TMP post-injury (Fig. 2D, E). Moreover, the expression of CXCR4 was assayed through immunohistochemical staining. The results indicate that the level of CXCR4 (brown) in corneal epithelium is reduced in the cornea on day 7 after TMP treatment (Fig. 2F). Taken together, these results demonstrate that TMP might exhibit an inhibitory effect on rat corneal NV through the down-regulation of CXCR4 expression.

### TMP inhibits bleomycin-induced pulmonary fibrosis

**Figure 3:**
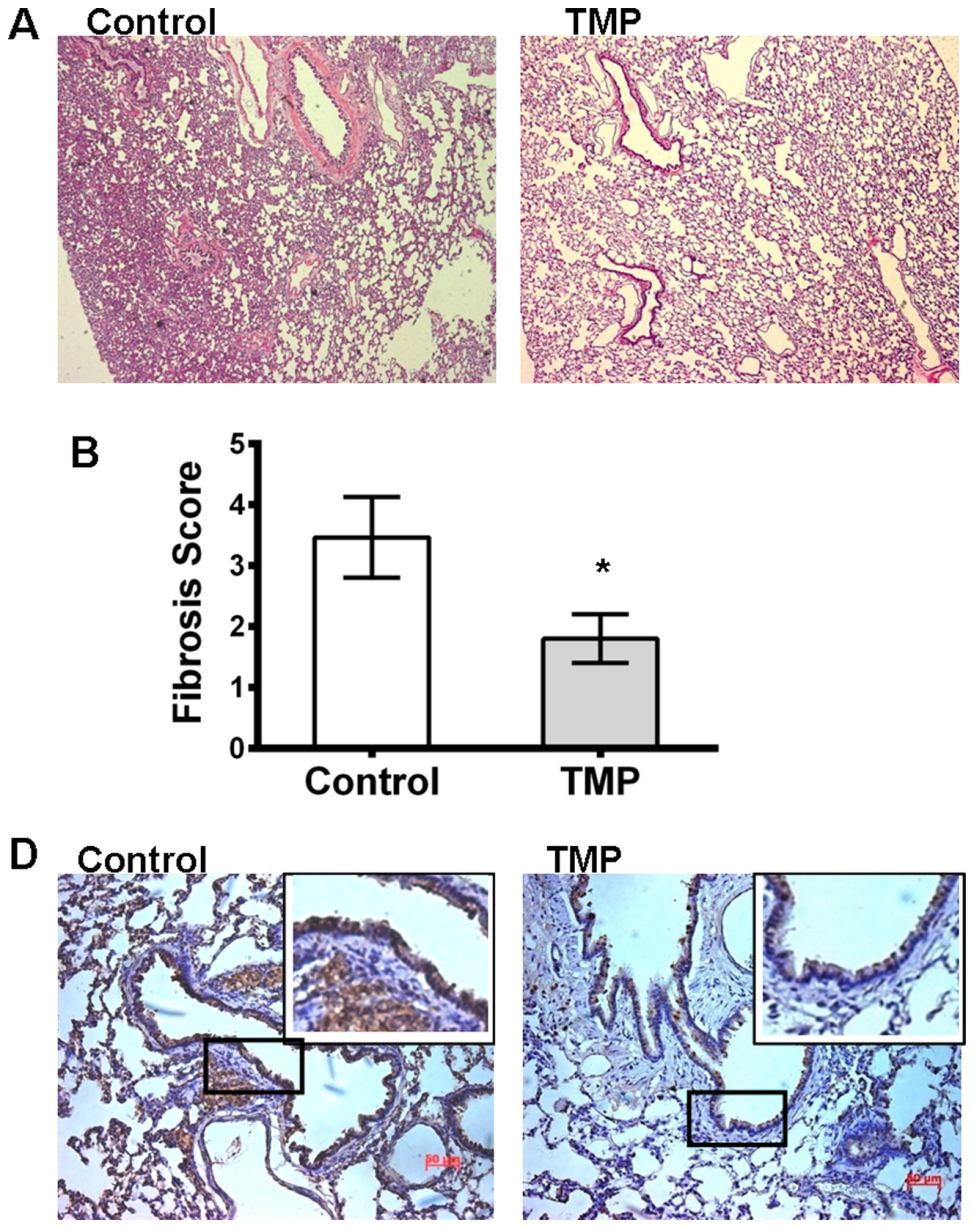
TMP inhibits bleomycin-induced lung fibrosis. (A) Hematoxylin and eosin staining shows that the extent of lung fibrosis in the TMP-treated group was ameliorated compared with the control group. (B) The scoring of fibrosis in lung sections was blindly determined through trichrome staining. (C) The expression of CXCR4 (brown) was assayed through immunohistochemical staining. Representative images show that the numbers of positive cells in lung tissue were notably reduced after TMP treatment. The asterisks represent statistically significant differences between the TMP treated and control groups (*p<0.005).

TMP also has a therapeutic effect on pulmonary fibrosis [28]. CXC chemokines, including CXCR4, play a pivotal role in the pathogenesis of this disease. Therefore, we speculated that TMP might regulate CXCR4 expression in pulmonary fibrosis. To examine this hypothesis, we used a bleomycin-induced lung fibrosis model to determine whether TMP affected lung fibrosis in vivo. The rats received intraperitoneal injections with 50-mg/kg TMP or saline at 1 d before bleomycin treatment and daily for 28 d thereafter. The agent/TMP was well tolerated in all rats. The lungs harvested on 28 d after bleomycin treatment were histologically analyzed and assayed for collagen content. Immunohistochemical staining showed that the extent of lung fibrosis in TMP-treated rats is greatly ameliorated and bleomycin causes marked increases in collagen deposition in the control rats (8.46±2.54 and 2.22±0.56, respectively) (Fig. 3A and B). Moreover, the expression of CXCR4 in lung tissue was investigated using immunohistochemistry. The results showed that the positive cells in lung tissue were notably reduced after TMP treatment (Fig. 3C).

### TMP decreases whole-blood viscosity and inhibits platelet aggregation

**Figure 4:**
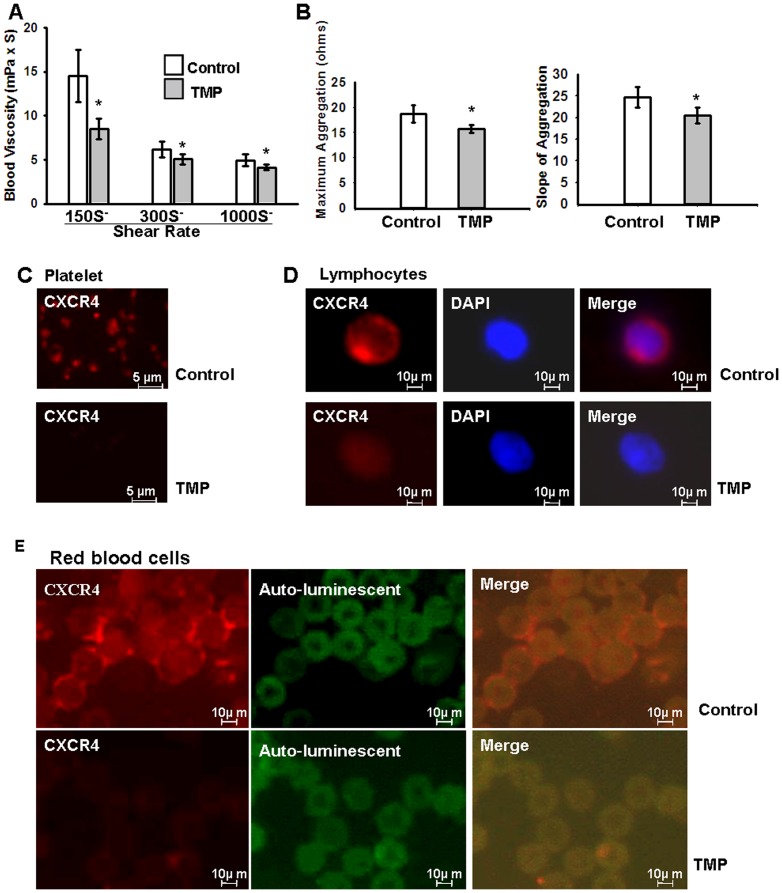
TMP affects whole-blood flow. Adult SD rats were randomized to treatment groups, which received daily i.p. injections of TMP or the control vehicle (normal saline) at a dose of 100 mg/kg in 500 µL for 3 days. (A) TMP decreases the whole-blood viscosity at shear rates of 150, 300, and 1000 sec-1 (14.50±2.49 vs. 8.54±1.15, 6.17±0.88 vs. 5.09±0.58, 4.96±0.64 vs. 4.15±0.26, respectively; *P<0.001; n = 6 rats/group). (B) TMP decreases ADP-induced platelet aggregation rate, the maximum platelet aggregation rate of (18.70±1.75 vs. 15.70±0.82), and the slope (24.70±2.42 vs. 20.50±1.87) (P<0.001; n = 6 rats/group). Platelets, lymphocytes and red blood cells were stained with anti-CXCR4 (red) primary antibodies, as described in Materials and Methods. The immunohistofluorescence analysis shows that CXCR4 expression of platelets (C), lymphocytes (D) and red blood cells (E) is significantly reduced after treatment with TMP.

TMP is widely used in the clinical therapy of ischemic cerebral and myocardial diseases in China, and whole-blood viscosity and platelet aggregation are used as prognostic indicators; the CXCR4/SDF-1 interaction is involved in these processes [24]–[25]. Therefore, we measured whole-blood viscosity and the platelet aggregation rate of SD rats treated with TMP or vehicle (normal saline) for 3 days. As shown in Fig. 4A, TMP significantly reduces whole-blood viscosity at shear rates of 300 and 1000 per second (14.50±2.49 vs. 8.54±1.15, 6.17±0.88 vs. 5.09±0.58, 4.96±0.64 vs. 4.15±0.26); in particular, shear rates of 150 per second are reduced by ∼41.1%. TMP attenuates the platelet aggregation rate (Fig. 4B), expressed as the maximum rate (18.70±1.75 vs. 15.70±0.82) and slope (24.70±2.42 vs. 20.50±1.87). The CXCR4/SDF-1 axis plays a key role in remodeling blood flow after infarction. Consistently, we observed that TMP significantly down-regulates the expression of CXCR4 in platelets (Fig. 4C), lymphocytes (Fig. 4D) and blood red cells (Fig. 4E).

## Discussion

In the present study, we demonstrated that TMP inhibits pathological angiogenesis, ameliorates pulmonary fibrosis, and decreases platelet aggregation. Interestingly, we also observed that the down-regulation of CXCR4 is involved in each of these processes, suggesting that the SDF-1/CXCR4 axis is a potential target of TMP.

As described in previous studies, the SDF-1/CXCR4 axis significantly promotes cell migration, enhances microvascular endothelial cell tube formation and tumor metastasis [16]-[18]. The data obtained in the present study demonstrates that TMP significantly decreases migration and attenuates tube formation in the human umbilical vein endothelial cell line ECV304 compared with the control (Fig. 1E, 1G). Significantly,TMP could counteract the effect of F-actin cytoskeleton remodeling like migratory structures formation induced by SDF-1 (Fig. 1H). Dynamic cytoskeleton rearrangement is crucial to cellular migration [29]. The pseudopodia protrusion formation immediately after the stimulated by SDF-1, contains a large number of filopodia-like structures and it was well inhibited by TMP and AMD3100, which may, at least in part, accounts for the anti-migration effect of TMP. Moreover, CXCR4 expression in ECV304 cells is notably down-regulated after TMP treatment. These results are consistent with previous reports, demonstrating that TMP significantly decreases CXCR4 expression in human retinoblastoma SO-Rb50 cells, cerebral neurocytes and rat C6 glioma cells [15], [26], [30]. More importantly, these results showed that corneal neovascularization is inhibited in alkali-injured cornea after TMP treatment. Semi-quantitative RT-PCR and staining also confirm that TMP effectively down-regulates CXCR4 expression in corneal tissue. These results are consistent with those from previous studies, suggesting that CXCR4 plays a key role in pathological angiogenesis. Lee et al. reported that the inhibition of CXCR4 could effectively reduce angiogenesis in alkali-induced corneal injury or laser-induced choroidal neovascularization models [31]–[32]. Therefore, the down-regulation of CXCR4 might be involved in the TMP-mediated inhibition of corneal neovascularization.

Some studies have suggested a different mechanism for the TMP-mediated inhibition of neovascularization. Liang et al. demonstrated that TMP prevented retinal neovascularization in an oxygen-induced retinopathy model [33], and these authors attributed this effect to the down-regulation of HIF-1α and VEGF. However, previous studies have indicated that CXCR4 positively regulates VEGF expression through the transcription factor Yin Yang 1 (YY1) and the Akt signaling pathway [34]–[35]. Moreover, in the present study, we demonstrated that the decrease in CXCR4 expression induced through TMP is 3.94-fold greater than the decrease in VEGF expression in C6 glioma cells [26]. Based on these data, we deduce that the down-regulation of CXCR4 induced through TMP treatment occurs prior to the reduction of VEGF expression.

The SDF-1/CXCR4 axis plays a critical role in the pathogenesis of pulmonary fibrosis [22]–[23]. Bone marrow-derived fibrocytes enter tissues following injury and contribute to fibrosis [36]–[37]. The trafficking mechanism of fibrocytes involves several chemokines/chemokine receptors, such as CXCL12/CXCR4, CCL21/CCR7, CCL2/CCR2 and CCL3/CCR5. In particular, the CXCL12/CXCR4 axis might play a major role in the regulation of fibrocytes migration both in vitro and in vivo [38]–[41]. CXCR4 expression immediately increased after bleomycin-induced injury and peaked at day 14. The fibrocytes expressing CXCR4 were collected in the context of mouse and human pulmonary fibrosis. The pharmaceutical interruption of this pathway, using AMD3100, results in the attenuation of pulmonary fibrosis through the inhibition of circulating fibrocyte migration. These data show that TMP significantly attenuates bleomycin-induced lung fibrosis. The immunofluorescence assay shows that CXCR4-positive cells in lung tissue are notably reduced after TMP treatment (Fig. 3C). Therefore, this finding also indicates that TMP can effectively inhibit lung fibrosis through the regulation of CXCR4.

TMP has been prescribed for cardiovascular diseases in China and is believed to be capable of attenuating platelet aggregation, preventing thrombus formation and thus improving microcirculation [7]–[8]. Consistent with previous reports, the data obtained in the present study also demonstrate that TMP significantly reduces whole-blood viscosity ∼41.1% at shear rates of 150 s-1 (Fig. 4A) and attenuates the platelet aggregation rate (Fig. 4B, 4C). Similarly, the expression of CXCR4 in platelets, lymphocytes and red blood cells in SD rats is significantly down-regulated after daily i.p. injections of TMP. These findings are consistent with those of previous reports. CXCR4 plays a critical role in optimal platelet activation and thrombus formation [42]–[43]. Blocking CXCR4 with the CXCR4 inhibitor AMD3100 effectively inhibits platelet accumulation, reduces thrombus formation and accelerates blood-flow restoration in vivo. Thus, these data indicate that TMP affects whole-blood flow through the down-regulation of CXCR4 expression.

In addition, the SDF-1/CXCR4 axis plays a critical role in the regulation of the immune system and is involved in the pathogenesis of several immunological conditions, such as WHIM syndrome, rheumatoid arthritis and lupus [44]–[45]. Blocking SDF-1/CXCR4 is a potential therapeutic strategy [19]. TMP has long been applied to treat diseases in China, and it has been suggested that TMP might regulate CXCR4 expression. Further investigation is required to confirm this hypothesis.

In summary, the results obtained in the present study suggest that regulating the SDF-1/CXCR4 axis through TMP might inhibit corneal neovascularization, attenuate pulmonary fibrosis, and improve microcirculation. In 2008, the FDA approved the first CXCR4 antagonist, plerixafor (AMD3100), for the mobilization of hematopoietic stem cells, and several other CXCR4 antagonists are currently in clinical trials for the treatment of cancer, HIV, and WHIM syndrome. While the long-term safety data for the first generation CXCR4 antagonists are not yet available, TMP has been shown to be a relatively safe agent in clinical practice for thousands of years. Therefore, the new insight provided by the present study will extend the application of TMP to clinical therapy in current medical practice.

## Materials and Methods

### Cell culture and chemical

The human umbilical vein endothelial cell line, ECV304, was obtained from the ATCC Collection (Manassas, VA) and maintained in DMEM (Invitrogen, USA) supplemented with 10% FBS containing 100 u/ml penicillin and 100 mg/ml streptomycin in a humidified atmosphere of 5% CO2 at 37°C. Tetramethylpyrazine Hydrochloride (TMP) was purchased from Harbin Medisan Pharmaceutical Co., China, and dissolved in normal saline to appropriate concentrations. Hence, normal saline was used as a control in all experiments. AMD3100 and SDF-1 was purchased from Sigma (St. Louis, MO).

### Western blot

The cells were grown to near confluency and lysed with RIPA buffer (50 mM Tris-HCl, pH 8.0, with 150 mM sodium chloride, 1.0% Igepal CA-630 (NP-40), 0.5% sodium deoxycholate, and 0.1% sodium dodecyl sulfate). Whole cell lysates were separated by sodium dodecyl sulfate/polyacrylamide electrophoresis, and transferred for 1 h to a nitrocellulose (PVDF) membrane; CXCR4 was detected using primary antibodies against CXCR4 (Santa Cruz Biotechnology Inc., CA) and a horseradish peroxidase-conjugated goat anti-rabbit secondary antibody (Santa Cruz Biotechnology Inc., CA). β-actin served as a loading control. The protein bands were detected using an enhanced chemiluminescence detection system.

### Cell migration assay

Wound healing assay was performed to quantify the rate of ECV304 cell migration. A total of 1×10^6^ ECV304 cells were seeded onto a 60-mm dish in the absence of TMP (100 µM), AMD3100 (10 µg/mL) or PBS for 48 h, and then a wound was created after manually scraping the cell monolayer with a p200 pipet tip. The initial wound quantification was performed on images collected at 0 hour after wounding, when the wound size had been stabilized. Further images were collected randomly in wound areas at 6 hours and 12 hours after wounding.

### Tubule formation in Matrigel

The human endothelial cell line, ECV304, was treated with TMP (100 µM), AMD3100 (10 µg/mL) and PBS respectively. At 48 hours after treatment, the cells were washed with PBS, detached using 1% trypsin, and seeded at 10^5^ cells/well onto Matrigel-coated (200 µl of 10 mg/ml) plates (BD Bioscience, CA). The plate was incubated at room temperature for 15 min and subsequently incubated at 37°C for 30 min to the Matrigel to polymerize. The cells were incubated for 24 h to facilitate capillary-like structure formation. The relative quantity of the tubules was quantified through Image Pro Plus software (Media Cybernetics Inc., CA).

### Animals and model of corneal alkali burn

A total of 30 adult Sprague Dawley rats weighing 250–300 g were obtained from the Laboratory Animal Centre, Southern Medical University (Guangzhou, China). All experimental procedures were approved through the Ethical Committee of Sun Yat-Sen University (2011-043). The rats were anesthetized with an intraperitoneal injection of 60 mg/kg of Nembutal and the topical administration of a drop of tetracaine. The corneal alkali-wound was made by placing a 1.5-mm diameter circular piece of filter paper, soaked in 1 N NaOH, in contact with the central cornea on the right eye for 40 sec. Immediately after alkali exposure, the ocular surface was rinsed with PBS for 60 sec. The rats were randomly divided into 2 groups (n = 15): Group 1 comprised rats subjected to alkali burn with normal saline treatment (10 µl, 4 times per day), and Group 2 comprised rats subjected to alkali burn with TMP treatment (1.5 mg/ml in a volume of 10 ml, 4 times per day). All eyes were observed on day 28 using slit lamp microscopy for the evaluation of corneal NV.

### Evaluation of NV in the cornea

Corneal NV (NV) was quantified as previously described, with modifications [46]. Briefly, an ophthalmologist blinded to the study conditions examined all eyes under a slit lamp microscope on days 1, 2, 5 and 8 after alkali burn. The corneal image was divided into 4 quarters. The vessel length of each quarter (Li, i = 1–4) was measured using a Vernier caliper. The corneal NV area (A) was calculated using the following equation: A = Σ_i_ = _1–4_ 3.1416×{R^2^−(R−Li)^2^} (R is the radius of the rat cornea. R = 3.5 mm, as calculated from the measurement of 15 rat corneas).

### Bleomycin-induced lung fibrosis

A total of 30 adult Sprague Dawley rats weighing 250–300 g were obtained from the Laboratory Animal Centre, Southern Medical University (Guangzhou, China). All experimental procedures were approved through the Ethical Committee of Sun Yat-Sen University (2011-043). The rats were anesthetized through isofluorane inhalation; the trachea was exposed using sterile techniques and 4 U/kg of bleomycin (Sigma) in 100 mL PBS or PBS vehicle was injected into the tracheal lumen. After inoculation, the incision was closed and the animals were allowed to recover. Each group contained 15 mice treated with 10 mg/kg of TMP or PBS intraperitoneally at 1 day before bleomycin treatment and daily for 20 days thereafter. The lungs were harvested at 20 days after bleomycin treatment and histologically analyzed through H&E staining, followed by imaging under a microscope [47].

### Effect of TMP on blood flow in SD rats

A total of 12 adult Sprague Dawley rats weighing 250–300 g were obtained from the Laboratory Animal Centre, Southern Medical University (Guangzhou, China). The rats were reared in the laboratory animal center of Zhongshan Ophthalmic Center, Sun Yat-Sen University. All experimental procedures were approved through the Ethical Committee of Sun Yat-Sen University (2011-043). The SD rats were randomized into treatment groups receiving daily i. p. injections of TMP or vehicle (normal saline) at a dose of 100 mg/kg in 500 µL for 3 days (6 rats for each group). The rats were anesthetized, and whole blood samples were collected from the femoral artery using a vacuum heparin anticoagulation collection tube. Immediately after arteriopuncture, the platelet aggregation rate was measured on a CHRONO-LOG, and whole-blood viscosity was measured on a Rheolog viscometer (Rheologics Inc, PA).

### Actin-polymerization assay

ECV 304 cells were seeded on cover slips in 6 well plates. The cells were serum starved overnight in the absence of TMP, AMD3100 or PBS, and then stimulated by 100 nM SDF-1 for 15 min. To terminate the reaction, the cover slips were washed gently with phosphate buffered saline (1×PBS), and then fixed in 4% paraformaldehyde for 15 min and washed three times with PBS. The slips were permeabilized for 10 min with 0.1% Triton X-100 and washed three times with PBS. Later they were stained with FITC–phalloidin (Sigma, St Louis, MO, USA), a water-soluble compound that selectively binds with F-actin, for 2 h. Finally, the cover slips were washed three times with PBS and mounted with Antifade. Photomicrographs were captured with a ZEISS LSM 510 confocal microscope at 488 nm to obtain digital images.

### Immunohistofluorescence assay

(1)ECV 304 cells were fixed with ice-cold 100% methanol for 15 min, and blocked with 10% normal goat serum for 30 min. Cells were then incubated overnight at 4°C with primary antibodies against CXCR4-fusin (1∶100 dilution, Santa Cruz Biotechnology Inc., CA). Alexa Fluor antirabbit 546 was used as secondary antibodies (1∶500 dilution, Santa Cruz Biotechnology Inc., CA), and nuclei were stained with DAPI. (2) Lung cryosections. Lung sections were fixed with 100% methanol at −20°C for 20 min and subsequently treated with a blocking solution (5% normal goat serum and 2% bovine serum albumin in PBS) for 30 min to prevent nonspecific antibody-antigen binding. CXCR4 expression was detected using a CXCR4-fusin antibody (1∶100 dilution; Santa Cruz Biotechnology Inc., CA) For the fluorescence visualization of antibody reactions, the primary antibodies were detected using secondary antibodies labeled with the fluorochromes Alexa Fluor 546 (1∶500; Invitrogen, CA), while the nuclei were detected with DAPI. (3) Lymphocytes, blood red cells and platelets. Lymphocytes, blood red cells and platelets were collected according to previously described procedures (24). The platelets were exposed to BSA-precoated glass coverslide chambers. The lymphocytes and blood red cells were exposed to 0.01% poly-L-lysine-precoated glass coverslides. The samples were fixed, permeabilized, and incubated with indicated primary antibodies against CXCR4 (1∶100; Santa Cruz Biotechnology Inc., CA). For fluorescence visualization of the antibody reactions, the primary antibodies were detected using secondary antibodies labeled with the fluorochromes Alexa Fluor 546 (1∶500; Invitrogen, CA). Photomicrographs were captured with an Axioplan 2-imaging microscope system (Carl Zeiss, Inc., Germany).

### Ethics statement

This study strictly adhered to the ARVO Statement for the Use of Animals in Ophthalmic and Vision Research and was approved and monitored by the Institutional Animal Care and Use Committee of Zhongshan Ophthalmic Center (Permit Number: SYXK (YUE) 2010-0058). The rats used in this study were from the Ophthalmic Animal Laboratory, Zhongshan Ophthalmic Center, Sun Yat-sen University. The animals were housed in an air-conditioned room with an ambient temperature of 16–26°C, a relative humidity of 40–70% and a 12-hour light-dark cycle with a daytime light intensity of approximately 200 lux. The animals were housed in rats cages with sufficient space and provided with a commercial mice diet. Animal health was monitored daily by the animal care staff and veterinary personnel. Rats were sacrificed by an intraperitoneal injection of Nembutal (P3761, 60 mg/kg) (Sigma, St. Louis, MO) before we harvested the eyes. All efforts were made to minimize suffering.

### Statistical analysis

All in vitro experiments were performed in triplicate. The data are expressed as the means+/2SE. The differences between the mean values were evaluated using the two-tailed Student's t-test (for 2 groups). All calculations and statistical tests were performed using Microsoft Excel 2003 (Microsoft, Redmond, WA) or SPSS 11.5 (SPSS, Chicago, IL) software. P<0.05 was considered significant for all analyses.
